# A Study of Empyema Cases at Camp Doniphan

**Published:** 1919

**Authors:** 


***A Study of Empyema Cases at Camp Doniphan.*** By Major C. B. Ingraham, Captain J. A. Roddy, and Captain J. D. Aronson. *Surgery, Gynecology and Obstetrics*, December 1918.

The cases classed as empyema are those in which pus and exudate with bacteria exist in the pleural cavity. From November 2, 1917, to May 4, 1918, there were 148 cases. Practically all men affected were in the 3rd decade of life, and the average period of military service before admission to the hospital was three months.

(1) *Classification.* There were 11 primary cases; the other 137 gave a history, and many showed signs of recent acute rhinitis, laryngitis, tonsilitis, or bronchitis. The onset was sudden in 47 o/o, gradual in 34 o/o, and insidious in 19 o/o of the cases diagnosed before death. There were 22 cases not diagnosed until autopsy. In general, the symptoms conformed with accepted descriptions; dyspnea was not a symptom of diagnostic value.

*Physical Signs.* Changes in contour and motion suggestive of fluid in the chest were observed in 5 o/o of the cases. Decrease in, or absence of, normal fremitus was detected in 76 o/o; increased fremitus in 9 o/o. Extreme dulness or flatness suggestive of fluid in the chest was elicited in 60 o/o of the cases; changes in percussion sound caused by change of patient's position were noted in 5 o/o. Hyper-resonance was elicited above the area of dulness in less than 1/2 the cases.

Pleural friction was heard in 15 o/o of the cases; vocal and breath sounds were diminished in 50 o/o, and absent in 10 o/o over the site of fluid; increased in 8 o/o; and intensity not noticeably altered in the remainder.

(2) *Course.* Empyema occurred secondary to demonstrable infection elsewhere in the body in more than 90 o/o of the cases. The course in favorable cases was that of a localized severe streptococcus infection, and in the fatal cases that of a streptococcus septicemia, usually with multiple foci of infection in adjacent organs. Leucocytosis showed only slight fluctuations throughout the disease. There was no distinctive difference in either the total or differential white cell count between mild and severe, or between fatal cases and recoveries.

(3) *Temperature.* The temperature curve bore no relation to the gravity of the case in most instances.

(4) *Character and Location of Exudate.* a) Cases yielding clear serous fluid the first two or three days, and thereafter cloudy serofibrinous, seropurulent fluid.

b) The fluid aspirated in several cases immediately before operation was cloudy serous, and, when the pleura was opened, frank pus was obtained.

c) Cases yielding apparently sterile fluid followed by fluid showing streptococci.

d) Cases in which 3 to 4 punctures were made before obtaining fluid.

e) Cases in which the exudate was sacculated; in 2 fatal cases the empyema was bilateral.

(5) *Complications.* The complications observed were pericarditis, peritonitis, pneumonia bronchitis and diphtheria. Postmcrtem findings indicate that grave pathological changes in the liver, spleen and kidneys were frequent complications; but such were not detected during the process of the disease.

(6) *Duration, Mortality and Morbidity.* In 4 cases operated on, recovery followed within an average period of about 30 days. The average duration before operation was reckoned as 22 days; after operation 56 days. In fatal cases, the duration before operation was 16 days, after operation 13 days.

MORTALITY

| | | |
|---|---|---|
| Total mortality . . . . . . . . . . . . . . . . . | 48.6 | o/o |
| Mortality, not operated. . . . . . . . . . . . . | 91 | o/o |
| " intercostal, incision and drainage. . . | 66.66 | o/o |
| " rib resection and drainage. . . . . . | 31.8 | o/o |

Convalescence over 3 months in all cases, and not one was found with full restoration to health.

(7) *Diagnosis.* Early diagnosis, essential to best results requires :

*a*) Careful physical examination of chest daily in all cases of streptococcal infection regardless of its location.

*b*) Immediate and repeated X-ray examination of chest in case of severe continuous pain in same.

*c*) Exploratory punctures with medium-sized trocart and canula.

*d*) Total differential leucocyte counts and thorough bacteriological investigation of fluid withdrawn.

(8) *Etiology.* The most important predisposing cause is a marked reduction in the normal immunity or resistance to disease from any cause, conspicuous among which have been : prolonged illness, undue exposure to cold, and unusual fatigue.

(9) *Treatment.* Absolute rest throughout the disease is an essential factor of recovery; milder cases of empyema tend to spontaneous recovery without medication, operation, or treatment other than careful nursing. In the great majority of cases, however, rib resection and drainage were required.

(10) *Anesthetic.* Nitrous oxide was considered the most satisfactory; ether is satisfactory and safe. The Carrel-Dakin solution gave no satisfactory results.

(11) *Laboratory Studies.* Blood cultures show the presence of organisms late in the disease in grave cases only, as a rule. A positive culture is a most unfavorable prognostic sign. The pleural exudate obtained from empyema cases, from which hemolytic streptococcus was recovered, showed a distinct difference in its physical characteristics, in the majority of cases, from the empyema fluid from which the pneumococcus was recovered.

(12) *Bacteriology.* The streptococci recovered showed the same morphological and cultural characteristics, but the irregularity of serological reactions makes it impossible to draw definite conclusions as to their belonging to the same group.

(13) *Postmortem Examination.* Postmortem findings in cases presenting both pneumonia and empyema caused by streptococci showed in most cases distinct lobar pneumonia, and, in a number of cases, the interstitial form of pneumonia, especially in the case of patients who had been ill for a long time.

